# Conservatism in Gynæcology

**Published:** 1888-06

**Authors:** A. Reeves Jackson

**Affiliations:** Professor of Gynæcology in the College of Physicians and Surgeons, Chicago; 271 Michigan Avenue


### The Medical Journal and Examiner.

VOLUME LVI. JUNE, 1888. NUMBER 6

### CONSERVATISM IN GYNÆCOLOGY.

BY. A. REEVES JACKSON, A. M., M. D.,*

Professor of Gynæcology in the College of Physicians and Surgeons, Chicago.

During the past quarter of a century, and especially during the past ten years, no department of medicine has shown greater activity or made greater advancement than that which embraces the abdominal and pelvic surgery of women. Many diseases which formerly baffled the utmost skill of the physician and surgeon, and which doomed the patient to a life of pain and misery or to certain death, are now treated with a highly satisfactory degree of success; and the lives that have been saved and made bearable by modern gynæcological methods may be counted by thousands.

It would be an invidous task to name those who have earned the world's gratitude by the part which they have taken in this noble work, and I shall not attempt it. But when we recall the fact that Sir Spencer Wells has performed over 2,000 ovariotomies; that Lawson Tait has opened the abdominal cavity over 2,000 times for various purposes; that many other operators, at home and abroad, number their gynæcological operations by hundreds upon hundreds, we cannot but be impressed by the great need for this work, and the incalculable benefit to suffering woman that has resulted from it. No words of praise can adequately meet the deserts of these apostles of humanity. But, as an obverse result of the great success which has been achieved in this direction, there has arisen a class of ambitious imitators, who, actuated by more conceit than conscientiousness, more zeal than judgment, are rapidly bringing discredit upon the honest work of others in the estimation of many who cannot, or at least do not, distinguish between the real and the sham, the true and the false. These persons are apparently afflicted with an uncontrollable itching for rapidly-acquired fame, and seem to think that they can achieve what they desire by beginning where their masters left off. With them, gynæcology no longer includes adequate effort for the cure of disease, it only signifies the art of cutting something out. It is not even necessary for their purposes that the organs and tissues invaded by them should be diseased; it is sufficient if only they have been left by their predecessors. In one instance within my knowledge when a spectator at an oöphorectomy suggested a doubt as to the necessity for the mutilation, the operator audaciously pointed out that the ablated organs were already in a state of incipient cystic degeneration—the

*Read before the Illinois State Medical Society May 15.

proof of the alleged fact consisting in the presence of Graafian follicles in various stages of development.

When a woman has an ovarian cystic tumor rapidly growing, and as certainly as a cancer, though more slowly, urging her towards death; or when she is suffering from the disgusting consequences of a urinary or fecal fistula; or from lacerations of the genitals so extensive as to impair or destroy function; or from various other manifest conditions of the pelvic or other organs attended by detectable alteration of structure, and which surely destroy health, comfort, or life, surgical aid is imperatively demanded and should be promptly rendered. But when operations involving dangers to life, such as laparotomy, are undertaken for the possible relief of vague nervous symptoms, hysteria, epilepsy not clearly of ovarian origin, insanity, or because there *is nothing else to do*, then such operations are improper and should not be made. When a man is nervous or irritable or insane does any one propose castration as a remedy? Would not such operation, although vastly less dangerous than the extirpation of the ovary, be regarded as malpractice? And when a corresponding operation is glibly proposed for a fretful, weak-backed, weak-minded, nervous woman, would not the opinion as to its necessity be greatly modified if the patient were a near relative—a mother, wife, sister, or daughter? And why? Because it is well known that such operations are not always, not even frequently, necessary; that they sometimes kill, and do not by any means generally cure. If any one finds himself in doubt as to the correctness of a conclusion in such a case, or as to his own honesty of purpose, let him take the case home to himself. If this were done there would be a smaller number of uterine appendages removed, a smaller number of hysterectomies performed. But, so long as the reputation of a gynæcologist, as such, is thought to depend upon the number of laparatomies he has done, rather than the number of patients he has cured and saved from mutilation, so long will the meetings of medical societies be enlivened by the presentation of platefuls of preserved ovaries and tubes.

And, in this connection, one cannot fail to be struck with the large percentage of recoveries which follow operations that are naturally dangerous. Whatever the nature of the specimen shown, however great may have been the difficulties of the operation, these latter, says the report, were all overcome, the operation was successful and "the patient doing well." The word "successful" in these cases means, usually, that the patient was not killed during the operation, and has no reference to the effect of the latter upon the condition for which it was done. These reports are sometimes made so soon after an operation that the patient has had no time to die unless from shock, ether-narcosis, or hæmorrhage, and then no report seems necessary. Once, at a meeting of a medical society at which I was present, a fibroid tumor of the uterus was exhibited. The uterus and appendages had been removed by laparatomy a few hours before. The patient was "doing well." Inasmuch as there did not appear to have been any symptoms sufficiently serious to warrant so dangerous an operation, I felt interested in learning the final result, which was that the patient continued to do well until she died six days later. This termination was not reported. An account of the case appeared subsequently in the published transactions of the society, with this heading, substantially, "Successful Removal of a Fibroid Tumor of the Uterus," etc.

This premature reporting of favorable cases (the others are frequently not reported at all) has the effect of misleading inexperienced persons who may feel tempted to repeat what seems to be so simple and beneficial.

It is to be considered, however, that unless such reports were made and specimens exhibited, the world would not know that this particular doctor had done such an operation; and while it is true that the world does not care for the knowledge, and would not be in any way worse for the lack of it, the doctor looks at it from a different standpoint—he wants his name to go into print. What the medical profession wants and needs from each worker in its ranks is a knowledge of the *results*—immediate and remote—of what he does, and such knowledge is always welcomed.

Compared with this unseemly eagerness to report cases, it is instructive and refreshing to read such a paper as that which was presented at the 12th annual meeting of the American Gynæcological Society, in September last, by Dr. Robert Battey, entitled "Battey's Operation, and its Matured Results." It covered the entire experience of the author, extending over a period of 17 years, and included 54 cases in which recovery took place. Of these, the number in which the patient was cured at once was very small. In the majority the patient passed through various climacteric disturbances, and the time which elapsed between the operation and the disappearance of abnormal symptoms varied from one to three, or even five years, thus showing the utter uselessness of reporting the results of removal of the ovaries within a few days or even months after the operation.

A correspondent of the *British Gynæcological Journal* (Nov. 1887) gave some of the results obtained by the late Professor Schroeder, who performed castration on some neurotic women. "In one case, two years after the operation, the woman's sexual appetite was 'completely dead,' but she was suffering from marked vaginismus. In the second case, one year after the operation, all the old cramps returned. The third case did well for some time, but five years after the operation she began to suffer from fainting fits."

Similarly, no good purpose is subserved by reporting a hysterectomy for cancer until sufficient time has elapsed to show the effect of the operation upon the disease.

Do you know to what extent and for what purposes this indiscriminate laparotomy is proposed and performed? Let me cite a few cases that have come under my own observation, all within less than a year.

About nine months ago a gentleman carried a woman in his arms into my office, and after placing her upon a lounge, said, abruptly, "Doctor, I want to know whether you can cure my wife without removing her ovaries." The question was more easily asked than answered, and I deferred giving an opinion until I had an opportunity of investigating the case. The woman was thirty years of age; she had been married eleven years, and had had two children and one miscarriage, the latter having occurred three years before. She was a well-formed, well-nourished woman of medium height, and had a wholesome visage. Menstruation was regular and painless, the functions of the bladder and rectum were normally performed, although there was a tendency to constipation; appetite fair, usually, but capricious. Physical examination revealed a slightly congested lower segment of the uterus, the cervix being somewhat club-shaped owing to a slight laceration of the os uteri, on the inner side of which was a superficial erosion, of no pathological importance. The ovaries and tubes could not be felt, and there was neither swelling nor tenderness in their neighborhood. The woman was fretful, irritable, and lazy, "Only this and nothing more." She had been under medical treatment for various ailments (which she probably did not have) during the last three or four years, and had spent the latter six months under the care of a gynæcologist in Chicago, who had used the regulation hot douches, glycerine tampons, massage, etc., without perceptible result. When the impatient husband asked whether nothing more, or nothing better, could be done, he was told that the only thing that offered relief was the removal of the ovaries. This suggestion did not meet with a favorable reception on the part of either husband or wife, and was therefore declined.

There was no pelvic disease present, and there was no further pelvic treatment. I will not trouble you with the details of what was done as it is not necessary for my purpose. Suffice it to say, that the patient left the city in a few weeks for her home. About two months ago the husband informed me by letter that his wife was pregnant, and still later, that she seemed very well and was able and willing to do all kinds of light work, and to take moderately long walks.

Again, a young woman, 19 years of age, had been suffering occasionally during several years from irritable bladder. She had been treated by a number of physicians with sometimes temporary benefit. Being finally referred to a gynæcological semi-specialist, he informed her that inasmuch as so many remedies had failed to cure her he would advise the removal of the ovaries. She still has them.

In a third case the patient was 22 years old. Menstruation commenced at 12, but had always been scanty and irregular, appearing at intervals of from six weeks to as many months. Her appetite was poor, complexion sallow, and her face affected with acne. She was advised to have her ovaries and tubes extirpated—with what object I cannot imagine unless it was to transfer them to a bottle or plate.

On May 1, her physician, in reply to a letter, wrote me as follows: "The soft rubber stem was retained for three months, which established her menstruation in much better condition than previous to its introduction—that is, as regards the quantity of the flow. I find she is in very good health compared with what she was last year."

Think of it! Women advised to have important organs removed, the mutilation involving dangerous operations, for the cure of a nervous affection of the bladder, for scanty menstruation dependent upon feeble general health, and for laziness!

At a meeting of the New York Pathological Society, held October 12, 1887, Dr. A. P. Dudley related the case of a woman 51 years of age who had ceased menstruation one year before. For several years she had suffered from pain in the region of the spleen, and an exploratory incision of the abdomen was recommended and performed. Nothing wrong was found with the spleen, and it was permitted to remain; but it was discovered that she had two ovaries with corresponding tubes, and although there was nothing abnormal about either of them they were promptly removed. The reason for this is obscure unless it be viewed in connection with the question gravely propounded by the operator at the close of his report, namely, "How much good can be done by the removal of the tubes and ovaries after the menopause?" The operation was done on the day immediately preceding the report, and although sufficient time had not elapsed to determine how much good it had done in this particular instance, it must have been gratifying to all concerned to know that the patient was "doing well." As there are still a good many women living who have passed the menopause, and who are still complete in their organization, there is a wide field from which to cull material that may afford an answer to this remarkable question.

Does not all this justify a remark recently made by Thomas Kieth (*British Medical Journal*), "In abdominal surgery responsibility seems to have become old-fashioned and gone out of date."

Dr. Horatio R. Bigelow presented a paper upon this subject to the Ninth International Medical Congress, in which, speaking of the dangerous tendency to operative measures, he says: "Who can begin to enumerate the number of cases in which the abdomen has been opened for supposed ovarian diseases, when not a trace of anything pathological was discoverable? Who will write the history of the cases in which perfectly healthy ovaries have been removed, as an offending cause, without one shadow of improvement in the general condition of the patient? A human being mutilated, deprived of its distinctive characteristics, and rendered miserable! A human life poised between earth and heaven to gratify the bad diagnosis, faulty pathology, or personal conceit of an irresponsible practioner! A human life *sacrificed to ambition upon the operating table!* Do you wonder, can you wonder, in the face of the grinning, horrid, damning facts, some of which are on record, and a host of which hide their ghastliness in dark places, that there should go out throughout the land a cry for conservatism?"

But, while the prevalent fashionable craze in radical gynæcology is laparotomy, there is another which is equally deserving of the bridle and bit. It is the extirpation of the cancerous uterus. The most ghastly chapter in the history of surgery is that which tells the story of the removal of the uterus for cancer. The operation has sacrificed more years of human life needlessly, uselessly, than any other surgical procedure; and it has perhaps never saved a life which was otherwise doomed. Its statistics, favorably distorted as they have been, show worse results, both immediate and remote, than do those of any other effective method of dealing with the disease. The operation has never been justified on the ground that it saved life; but other grounds of justification have been urged which are at least curious and interesting, if nothing else. In the discussion of the subject at the recent International Medical Congress, one of the participants (Dr. Dirner, of Buda-Pesth) held that the operation was a good thing for everybody concerned—for the operator, (whom he praised for his work), for such of the patients as recovered, and for even those who died, for the latter were thus enabled to avoid the painful and disgusting accompaniments of the dreadful malady. Dr. Dirner here struck upon the only firm basis of justifiability, for, if the operation be proper at all, it must be on the ground that it lessens suffering by shortening life. This is legitimate reasoning because it accords with the facts.

Another gentleman,* who had previously published a report of sixty-six operations by American surgeons, with a mortality of nearly 35 per cent., while admitting that such results "would seem to indicate that the operation is an unjustifiable one," excused the unfortunate showing on the ground that many of the operators had had no experience whatever, and others very little in this direction, and *that nothing better therefore should be expected of them!* But, while thus admitting their incompetency he had no word to speak in condemnation of their selfish temerity in thus tampering with the lives of the victims of their ambition. On the contrary, he recommended that, as "practice makes perfect," these men and such as these should go on acquiring experience, because, he added, "Americans have not yet had a fair trial." Here, then, is a new ground of justification —an ethical one—namely, that surgeons ought to learn how to do the operation even though they sacrifice the lives of one-third of their patients in getting the knowledge.

If the soundness of this doctrine were admitted, I fear that a surgeon who felt very eager to remove a uterus might not be over-particular as to the accuracy of the diagnosis. Indeed, as an evidence that this fear is not a fanciful one, I may mention that at a meeting of the Obstetrical Society of New York, held October 4, 1887, Dr. Tuttle cited two cases within his own knowledge in which the uterus had been unnecessarily removed for supposed malignant disease. In one case, in which the patient recovered, the diagnosis of sarcoma was disproved by the microscope; in the other, which terminated fatally, the hæmorrhage was found to have been due to a submucous polypus.

A surgeon who performs a rational operation, however dangerous it may be, having

* Dr. A. Palmer Dudley, *New York Medical Journal*, July 9 and 16, 1887.

for its object the saving or prolongation of a human life, does a commendable act. But if, after such operation has been repeated hundreds of times, by himself and others equally skillful, and the results have shown it to be useless, or still worse, injurious, he should still persist, he ought not to be surprised at drawing censure upon himself, and that doubts should arise among his brethren as to the purity of his motives, or, at least, as to the correctness of his judgment. There was a time when the extirpation of a cancerous uterus was unquestionably justifiable, because, theoretically it offered a better chance for ultimate recovery than any method of treatment hitherto employed; and its greater immediate mortality as compared with that of other methods was not, alone, a valid objection. But when, after hundreds of trials, it was shown to be not only more fatal, but to be followed by no better, or even worse remote results, then, according to all candid reasoning, it should have been abandoned. But it has not been abandoned, and will not be so long as the honest and thoughtful element in the medical profession is not animated by a more actively conservative spirit than it has yet shown. Thus far it has been content to look on and keep silent. The men who are engaged in this objectionable work are the radicals in the gynæcological ranks. Not very long ago they were slitting up wombs; then they began to sew them up; they advised every woman who had a fibroid in the uterus, however harmless it might be, to have it removed—the tumor, or uterus, or both—and they did it, too, whenever they found a consenting victim.

The evils to which I have called attention are, in my judgment, very great. I fear they are growing. A woman has an inalienable right to all the organs with which she has been endowed. She should only be deprived of any of them when it is clear that through disease or faulty function, *otherwise incurable*, her health is seriously impaired or destroyed, or her life imperiled; and only then when the deprivation reasonably promises relief. To those who, from selfish, reckless, or other improper motives, would remove from a woman any of her peculiar organs, I say, in the name of womanhood, in the name of common honesty, in the name of humanity, hands off!

271 MICHIGAN AVENUE.

